# Menin inhibitors in pediatric acute leukemia: a comprehensive review and recommendations to accelerate progress in collaboration with adult leukemia and the international community

**DOI:** 10.1038/s41375-024-02368-7

**Published:** 2024-08-23

**Authors:** Branko Cuglievan, Hagop Kantarjian, Jeffrey E. Rubnitz, Todd M. Cooper, C. Michel Zwaan, Jessica A. Pollard, Courtney D. DiNardo, Tapan M. Kadia, Erin Guest, Nicholas J. Short, David McCall, Naval Daver, Cesar Nunez, Fadi G. Haddad, Miriam Garcia, Kapil N. Bhalla, Abhishek Maiti, Samanta Catueno, Warren Fiskus, Bing Z. Carter, Amber Gibson, Michael Roth, Sajad Khazal, Priti Tewari, Hussein A. Abbas, Wallace Bourgeois, Michael Andreeff, Neerav N. Shukla, Danh D. Truong, Jeremy Connors, Joseph A. Ludwig, Janine Stutterheim, Elisabeth Salzer, Kristian L. Juul-Dam, Koji Sasaki, Kris M. Mahadeo, Sarah K. Tasian, Gautam Borthakur, Samantha Dickson, Nitin Jain, Elias Jabbour, Soheil Meshinchi, Guillermo Garcia-Manero, Farhad Ravandi, Eytan M. Stein, E. Anders Kolb, Ghayas C. Issa

**Affiliations:** 1 Department of Pediatrics, The University of Texas MD Anderson Cancer Center, Houston, TX USA; 2 Department of Leukemia, The University of Texas MD Anderson Cancer Center, Houston, TX USA; 3 Department of Oncology, St Jude Children’s Research Hospital, Memphis, TN USA; 4 Cancer and Blood Disorders Center, Seattle Children’s Hospital, University of Washington, Seattle, WA USA; 5 Princess Maxima Center for Pediatric Oncology, Utrecht, the Netherlands; Pediatric Oncology, Erasmus MC-Sophia Children’s Hospital, Rotterdam, the Netherlands; The Innovative Therapies for Children with Cancer Consortium, Paris, France; 6 Department of Pediatrics, Harvard Medical School, Boston, MA USA; 7 Department of Pediatric Oncology, Children’s Mercy, Kansas City, MO USA; 8 Division of Transplant and Cellular Therapy, Loma Linda University School of Medicine, Loma Linda, CA USA; 9 Department of Stem Cell Transplantation and Cellular Therapy, The University of Texas MD Anderson Cancer, Houston, TX USA; 10 Department of Pediatrics, Memorial Sloan Kettering Cancer Center, New York, NY USA; 11 Department of Sarcoma Medical Oncology, The University of Texas MD Anderson Cancer Center, Houston, TX USA; 12 Princess Máxima Center for Pediatric Oncology, Utrecht, the Netherlands; 13 Department of Pediatrics and Adolescent Medicine, Aarhus University Hospital, Aarhus, Denmark; 14 Division of Pediatric Transplantation and Cellular Therapy, Duke University, Durham, NC USA; 15 Department of Pediatrics and Abramson Cancer Center, University of Pennsylvania School of Medicine, Philadelphia, PA USA; 16 Clinical Research Division, Fred Hutchinson Cancer Research Center, Seattle, WA USA; 17 Department of Leukemia, Memorial Sloan Kettering Cancer Center, New York, NY USA; 18 Moseley Institute for Cancer and Blood Disorders, Nemours Children’s Health, Wilmington, DE USA

**Keywords:** Leukaemia, Differentiation

## Abstract

Aberrant expression of *HOX* and *MEIS1* family genes, as seen in KMT2A-rearranged, NUP98-rearranged, or NPM1-mutated leukemias leads to arrested differentiation and leukemia development. *HOX* family genes are essential gatekeepers of physiologic hematopoiesis, and their expression is regulated by the interaction between *KMT2A* and menin. Menin inhibitors block this interaction, downregulate the abnormal expression of MEIS1 and other transcription factors and thereby release the differentiation block. Menin inhibitors show significant clinical efficacy against KMT2A-rearranged and NPM1-mutated acute leukemias, with promising potential to address unmet needs in various pediatric leukemia subtypes. In this collaborative initiative, pediatric and adult hematologists/oncologists, and stem cell transplant physicians have united their expertise to explore the potential of menin inhibitors in pediatric leukemia treatment internationally. Our efforts aim to provide a comprehensive clinical overview of menin inhibitors, integrating preclinical evidence and insights from ongoing global clinical trials. Additionally, we propose future international, inclusive, and efficient clinical trial designs, integrating pediatric populations in adult trials, to ensure broad access to this promising therapy for all children and adolescents with menin-dependent leukemias.

## Introduction

The onset, spectrum, and genetic complexity of pediatric cancers differ from cancer in adults [1]. Chromosomal rearrangements resulting in the formation of fusion proteins and dysregulation of essential transcription factors play a vital role in the pathogenesis of several pediatric cancers, including acute leukemia [2].

**Fig. 1:**
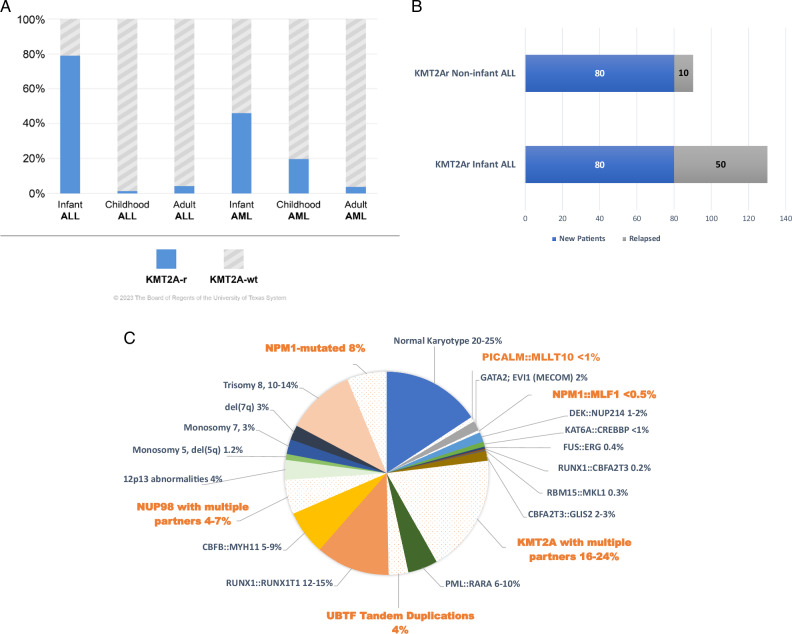
Targetable mutations for menin inhibitors across age distribution in ALL and AML. **A** Frequency of *de novo KMT2A*-r leukemias by age. (Data are from a series of 2381 prescreened acute leukemia patients analyzed by the Frankfurt Diagnostic Center of Acute Leukemia) [41]. **B** Estimated annual incidence of newly diagnosed and relapsed *KMT2A*-r ALL among infants and non-infants in COG studies. Note the distinct rates of relapse, ALL, acute lymphoblastic leukemia. **C** Breakdown of molecular subtypes of pediatric AML. Highlighted subtypes are associated with aberrant HOX/MEIS1 expression.

Chromosomal rearrangements involving the lysine (K)-specific methyltransferase 2 A (*KMT2A*, located on chromosome 11q.23.3, and previously known as mixed lineage leukemias or MLL) are associated with both de novo and therapy-induced infant and pediatric acute leukemias (Fig. 1A) [3]. The MLL1 protein is part of a large chromatin-modifying complex consisting of more than 30 proteins that regulate transcription through acetylation and methylation of histones. Wildtype KMT2A is a major regulator of hematopoiesis and embryonic development, through regulation of HOX gene expression (HOXA9 and MEIS1 in particular).

*KMT2A*, which plays a crucial role in embryonic development and hematopoiesis, is located at the 11q23 locus and encodes MLL1, a 430-kDa protein [4]. MLL1 undergoes proteolytic cleavage by taspase 1, which splits the protein into two fragments, MLL1-N and MLL1-C, between the plant homeodomain (PHD) fingers and the transactivation domain [5–8]. These subunits serve different functions: MLL1-N interacts with chromatin and acts as a transcriptional regulator, while MLL1-C is responsible for transactivation and methyltransferase activity. Menin binds to MLL1 in the Menin binding motifs (MBM), in the N-terminus of the MLL1 [9–11].

**Fig. 2:**
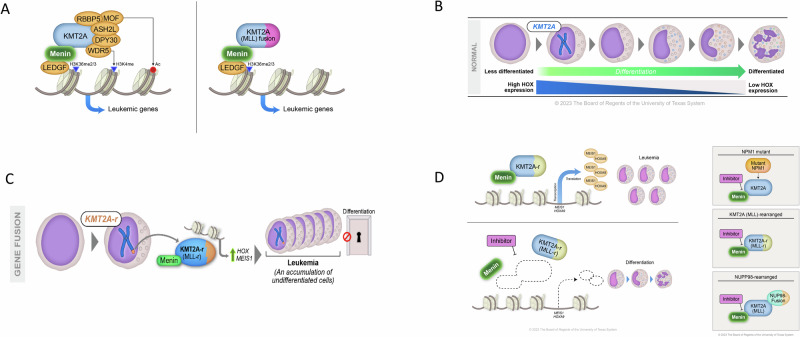
*KMT2A* rearrangement leads to leukemogenesis. **A** Interaction among KMT2A (MLL1), menin, and LEDGF. Menin binding sites are preserved during *KMT2A* rearrangement. **B** Cell differentiation and the gradual decrease of the HOX program. **C** The MLL1 fusion protein (MLL1-FP) leads to the overexpression of HOX cluster genes and *MEIS1*, contributing to the uncontrolled proliferation of undifferentiated precursor cells. **D** Novel synthesized menin inhibitors disrupt the menin-KMT2A interaction, effectively preventing the binding of MLL1-FP to promoter sites. This disruption halts the aberrant expression of HOX genes and *MEIS1*, resulting in the release of the differentiation block. Menin inhibitors disrupt NUP98 fusion occupancy at chromatin sites and disrupt MLL-NPM1 activity. This induces differentiation and reverses leukemogenesis by downregulating *MEIS1* expression.

*MEN1* is a tumor suppressor gene located on chromosome 11q13. The menin protein, the product of *MEN1*, is expressed at varying levels in multiple tissues. It is believed to function as a scaffold protein and interact with cell signaling and gene regulators. Unique among proteins, menin binds to DNA through nuclear localization sequences in the C-terminal region [12–14]. Once bound, menin links MLL1 and lens epithelium-derived growth factor (LEDGF), a chromatin-binding protein (Fig. 2A) [15, 16]. Because LEDGF contains the highly conserved motif PWWP, it serves as a modified-chromatin reader by recognizing both DNA and histone-methylated lysine [17]. In particular, the MLL1-menin-LEDGF ternary complex has a critical role in regulating the expression of *HOX* genes, such as the leukemogenic homeobox A9 gene *HOXA9* and its co-factor myeloid ecotropic virus insertion site 1 (*MEIS1)* [15, 18–20].

*HOXA9* is highly expressed in hematopoietic stem cells and early progenitors, and this expression gradually decreases as cells differentiate (Fig. 2B) [21]. Chromosomal translocations involving *KMT2A* result in a newly constituted MLL1 fusion protein (MLL1-FP) [22]. The MLL1-FP operates within a multi-protein complex that includes co-factors such as menin, DOT1L, LEDGF, and the super elongation complex [23–26]. Together with these co-factors, MLL1-FP drives the abnormal overexpression of *HOX* cluster genes and *MEIS1*, leading to the deregulation of normal hematopoiesis [6]. The overexpression of both *HOX* cluster genes and *MEIS1* promotes the uncontrolled proliferation of undifferentiated precursor cells, contributing to the development of acute leukemia. (Fig. 2C, D).

*KMT2A*-rearranged (*KMT2A*-r) leukemias are associated with leukocytosis at initial clinical presentation, resistance to standard chemotherapy, and higher relapse rates [9, 27–32]. More than 135 fusion partners for *KMT2A* have been identified in acute lymphoblastic leukemia (ALL) and acute myeloid leukemia (AML) [3, 19, 29]. While *de novo KMT2A-*r leukemias are generally associated with a poor prognosis in adults, prognosis in children is more variable [29, 32–35]. In addition, therapy-related leukemias that arise from exposure to topoisomerase II inhibitors are characterized by *KMT2A* rearrangements and are associated with treatment failure [36]. *KMT2A*-r ALL has a typical immunophenotype of CD34^+^CD19^+^ pro-B or early pre-B cells with variable CD10 expression but can rarely present as T-cell ALL [37]. The disease may also have co-expression of myeloid markers such as CD15 and CD65, which led to this subtype being known as mixed-lineage leukemia [38, 39]. Lineage plasticity, characterized by switches between lineages, poses a significant therapeutic challenge in patients with *KMT2A*-r ALL [38–40]. Despite intensified treatments and numerous therapeutic advancements in leukemia, many children with KMT2A-r ALL or AML continue to have poor outcomes. Given the dismal prognosis, particularly after relapse, there is an urgent need to enhance our scientific understanding of these leukemias and develop novel, effective therapies.

Extensive genetic and biological investigations recently revealed opportunities for precision medicine-based treatments that target the distinct vulnerabilities of acute leukemias with *KMT2A* rearrangements or other genetic alterations that menin inhibitors could target. This article, in addition to briefly reviewing common pediatric leukemias and their treatment challenges, provides an overview of the use of menin inhibitors in treating pediatric leukemia.

## Menin-dependent subtypes of childhood leukemias

While acute leukemias with *KMT2A* rearrangements occur in patients of all ages, the prognosis varies significantly among pediatric patients based on the specific leukemia subtype, age at diagnosis, and the fusion partner associated with *KMT2A*.

### *Pediatric KMT2A*-r leukemia by subtype

#### Infant KMT2A-rearranged ALL

*KMT2A*-rearrangements occur in approximately 75% of infants with ALL, and are associated with early treatment failure and poor outcomes (Fig. 1B) [41–43]. In trials performed by large cooperative groups, the 5-year event-free survival (EFS) was <40% for *KMT2A*-rearranged patients versus around 75% in patients with KMT2A-wildtype infant ALL [27, 35, 44]. *KMT2A*-r infant ALL harbors myeloid features, therefore in many protocols low dose and high dose cytarabine are added to an ALL backbone. However, when randomizing a myeloid consolidation to a lymphoid consolidation there was no difference in outcome [27, 33]. Only patients with a poor response to induction therapy seemed to benefit from myeloid-directed consolidation courses [27, 33]. Incorporating one course of blinatumomab after induction, into the Interfant-06 chemotherapy backbone, significantly improved outcome, with a 2-year disease-free survival of 81.6% compared to 49.4% in the Interfant-06 trial, which will now be tested prospectively in the new Interfant-21 protocol (NCT05327894). Importantly, this combination maintained a favorable safety profile in infants with *KMT2A*-r ALL [45]. In the Interfant-99 study, consolidation with hematopoietic stem cell transplantation (HSCT) was associated with a significant difference in disease-free survival (adjusted by waiting time to HSCT) between those who received HSCT and those who received chemotherapy only. However, the advantage was restricted to a subgroup with additional unfavorable prognostic features including age less than 6 months and either poor response to steroids at day 8 or high leukocyte count at presentation [46, 47]. Early relapse limited many patients’ eligibility for HSCT. The overall survival (OS) rate after relapse is 20% [48]. Interfant-21 incorporates HSCT for high-risk patients, and medium-risk infants with inadequate MRD response after induction. HSCT could improve survival but increases the risk of toxicities. Altogether, the prognostic considerations and therapeutic approaches for infants with *KMT2A*-r ALL remain illustrative that specific factors, including leukemia subtype, age at the time of diagnosis, MRD response, and the particular fusion partner associated with *KMT2A*, impact outcomes [32, 33, 35, 40, 42].

#### Childhood (non-infant) KMT2A-rearranged ALL

*KMT2A*-r ALL accounts for approximately 2% of ALL cases among children older than 12 months and the incidence of relapse is lower than in infants (Fig. 1B) [49, 50]. *KMT2A*-r ALL has a typical immunophenotype of CD34^+^CD19^+^ pro-B or early pre-B cells with variable CD10 expression but can rarely present as T-cell ALL [37]. The disease may also have co-expression of myeloid markers such as CD15 and CD65, which led to this subtype being known as mixed-lineage leukemia. Lineage plasticity, characterized by switches between lineages, poses a significant therapeutic challenge in patients with *KMT2A*-r ALL [38–40].

Attarbaschi et al., analyzed data from 629 patients and found that the most frequent translocations were t(4;11)(q21;q23) (51.5%), t(11;19)(q23;p13.3) (20.0%), t(9;11)(p21_22;q23) (14.3%), t(6;11)(q27;q23) (3.8%), and t(10;11)(p12;q23) (2.6%) [31]. Patients with these translocations have diverse characteristics and early treatment responses, indicating variations in therapy sensitivity. For example, patients with t(4;11) translocations had the highest rate of high MRD disease at early treatment response evaluation, the lowest 5-year EFS rate (64.8%), and the highest risk of relapse. For the whole *KMT2A*-r population, the 5-year EFS rate was 69.1% [31]. In a separate study, Pui et al. found that the 5-year EFS rate for the entire *KMT2A*-r group was 59.3%, significantly lower than the 87.3% observed in patients without KMT2A-r [40].

#### KMT2A-rearranged AML

*KMT2A* rearrangements are detected in 15–25% of pediatric AML patients (Fig. 1C) [51–55]. Patients often present with high-risk clinical characteristics, including a high white blood cell count and extramedullary involvement, as well as a peak in incidence at a young age (infants) [32]. Studies have shown no significant improvement of outcome metrics over time in children with *KMT2A-r* AML who remain associated with intermediate or inferior 5-year EFS and OS rates of around 45% and 60%, respectively [32, 51]. However, the prognostic value of *KMT2A* rearrangement is highly dependent on the fusion partner gene. In a retrospective international study of 1130 pediatric patients with *KMT2A*-r AML, patients harboring abnormalities in 6q27 (KMT2A::AFDN), 4q21 (KMT2A::AFF1), 10p12 (KMT2A::MLLT10), 10p11.2 (KMT2A::ABI1), and 19p13.3 (KMT2A::MLLT1) were categorized into the high-risk group, with all remaining cases allocated to the non–high-risk group [32]. The high-risk cohort exhibited lower EFS rates (30.3% for high risk vs. 54.0% for non-high risk; *P* < 0.0001), higher cumulative incidence of relapse (CIR) (59.7% vs. 35.2%; *P* < 0.0001), and worse OS (49.2% vs. 70.5%; *P* < 0.0001). Until recently, no specific agent or regimen of conventional therapy had shown evidence of particular efficacy in KMT2A-r AML. However, the Children’s Oncology Group (COG) AAML0531 trial showed that the addition of gemtuzumab ozogamicin, an antibody-drug conjugate targeting CD33, to conventional chemotherapy improved the outcomes of pediatric patients with *KMT2A*-r disease to a 5-year EFS of 48% [29].

Myeloid neoplasm post-cytotoxic therapy (MN-pCT) in children and adolescents is a rare but devastating malignancy, caused by chemotherapy, radiotherapy, previous malignancies or immunosuppressive treatment [36]. The overall prognosis is generally poor, and curative intent often requires allogeneic HSCT. Up to 70% result from topoisomerase II inhibitors and exhibit translocations in chromosome bands 11q23 or 21q22. They often develop within 2 or 3 years of the initial cytotoxic therapy, and sometimes within 12 months [36, 56–58]. In one study of 145 pediatric t-AML patients seen from 1993 to 2018, the 5-year OS rate was 28% for the entire cohort [59].

### *NUP98*-rearranged AML

Approximately 4–7% of pediatric patients with AML have disease characterized by (or driven by) NUP98 rearrangements (*NUP98*-r AML) (Fig. 1C); it is most common in male patients who are not infants (median age, 11.8 years) [60–62]. A collaborative study conducted by the COG and European AML study groups focused on defining the biological and clinical characteristics of patients with AML with *NUP98::KDM5A* rearrangements, excluding those with acute megakaryoblastic leukemia. The 5-year EFS and OS rates were 29.6% and 34.1%, respectively, whereas those without rearrangements were 47.0% ± 2.1% (*P* = 0.005) and 63.7% ± 2.1% (*P* ≤ 0.001), respectively [63, 64]. The study also revealed that of the 7 patients with *NUP98::KDM5A* rearrangements who underwent HSCT during their first CR, 5 had relapse, and 4 of those patients subsequently died [63]. Similar findings were observed in the ELAM02 clinical trial, in which the 3-year EFS rate of patients with *NUP98* rearrangements was considerably lower (10%) than that of patients with wildtype *NUP98* (60.5%) [65, 66]. Barresi et al. conducted a study to identify NUP98r AML (particularly NUP98::NSD1) primary induction failure associated genes, identifying 9 potential candidates, including SPINK2 and CDCP1 [64].

### NPM1-mutant AML

In pediatric AML, NPM1 mutations occur in 8–10% of cases and approximately 25% of those with a normal karyotype. (Fig. 1C) [67, 68]. Children with this genotype have high response rates, with reported 5-year EFS of 50%, without needing allogeneic HSCT [69, 70]. Those with concurrent *NPM1* and *FLT3* mutations also have favorable outcomes and may benefit from adding an *FLT3* inhibitor [69, 71].

### AML with *UBTF* tandem duplications

Recent studies revealed tandem duplications of UBTF (UBTF-TDs) in pediatric AML, constituting 4% of new cases and 9% of relapses [72–74] (Fig. 1C). UBTF-TDs co-occur with FLT3-ITDs and WT1 mutations, with normal karyotype or isolated trisomy 8 [75]. They are mutually exclusive with NPM1 mutations and recurrent fusions, and activate HOXA/HOXB cluster genes [74, 76]. Associated with poor outcomes and residual disease post-chemotherapy, UBTF-TDs may signify a high-risk pediatric AML subtype [72, 74, 77].

### Targeted therapies for menin-dependent childhood leukemias

Menin inhibition leads to global displacement of Menin from chromatin. However, the binding of MLL1 or its fusion proteins to chromatin is selectively impaired at a small subset of highly Menin-dependent target genes [18, 20, 78, 79]. Consequently, the aberrant expression of *HOX* and *MEIS1*, which contributes to the differentiation block in leukemic cells, is halted (Fig. 2D).

Targeting the interaction between menin and MLL1 has also shown benefits in leukemias with other genetic mutations. Menin inhibitors have demonstrated efficacy in preclinical models of leukemia with *NUP98* fusion [80, 81]. Treatment with menin inhibitors disrupted *NUP98* fusion occupancy at specific chromatin sites and improved survival in mouse models engrafted with *NUP98* fusion–bearing cells (Fig. 2D). In *NPM1*-m disease, menin inhibitors can disrupt the binding of cytoplasmic NPM1 (NPM1c) to MLL targets and disrupt MLL-NPM1 activity, inducing differentiation and reversing leukemogenesis through the downregulation of *MEIS1* [82] (Fig. 2D). In addition to *KMT2A*-r, *NUP98*-r, and *NPM1c* AML, several other AML subtypes, such as *PICALM*::*MLLT10* or *DEK*::*NUP214* fusions, are characterized by enforced HOXA and/or MEIS1 upregulation and thus potentially sensitive to menin inhibitors [83, 84]. Barajas et al. recently demonstrated that UBTF-TD and KMT2A/menin complexes co-occupy genomic targets that are dysregulated in *UBTF*-TD-positive AML. In addition, this study also showed that *UBTF*-TD-positive AML is sensitive to menin inhibition [85].

### Agents in clinical trials

#### Revumenib

The AUGMENT-101 trial (NCT04065399) is the first phase 1/2 clinical trial of an oral menin inhibitor, and included pediatric patients early in the development of the drug [82]. The trial is evaluating the safety and efficacy of revumenib (SNDX-5613, Syndax Pharmaceuticals) in adult and pediatric patients with relapsed/refractory *KMT2A*-r, or *NPM1*-m AML or ALL. Since revumenib is metabolized by CYP3A4, the study includes two separate dose-expansion groups to investigate the impact of potent CYP3A4 inhibitors, specifically antifungal prophylaxis, on the treatment. Patients in both arms receive oral revumenib every 12 h without interruption for 28-day cycles. In its early stages, the trial was open to all patients with relapsed leukemias, irrespective of cytogenetic and mutational profile. The trial was later amended to limit enrollment to patients with *KMT2A*-r or *NPM1*-m leukemia because preclinical data demonstrated the efficacy of targeting the *KMT2A*-menin interaction. As a result, 88% of the study population had *KMT2A*-r or *NPM1*-m leukemia at the time of the reported data analysis.

By July 24, 2023, 132 patients, ranging from 0.8 to 82.0 years old, diagnosed with relapsed or refractory (R/R) acute leukemia, were enrolled in the phase 1 study. Among them, 77 patients with R/R KMT2A-r acute leukemia received treatment with revumenib across six dose-escalation arms, including 15 pediatric patients (11 AML and 4 ALL). These patients had undergone a median of 3 prior lines of therapy, and 46.8% had a history of prior HSCT [86].

Results from Phase 1 in the R/R KMT2A-r subgroup showed a combined complete response (CR) and complete response with a partial hematologic recovery (CR+CRh) rate of 31.2% and an overall response rate (ORR) of 64.9%, with 38% proceeding to HSCT. In the pediatric subgroup, 10 patients demonstrated a positive response (1 CR, 2 CRh, 2 CR with incomplete platelet recovery, and 5 morphologic leukemia-free state), 4 of whom proceeded to HSCT. In the overall population of 132 patients, 25% experienced grade 3 or higher treatment-related adverse events (TRAEs), including grade 3 QTc prolongation in 8.3% and grade 3 differentiation syndrome (DS) in 2.3%. A total of 10.6% of patients discontinued revumenib due to TRAEs. The recommended phase 2 dose (RP2D) based on pharmacokinetics, clinical activity, and safety data was established as 276 mg q12h (or 160 mg/m^2^ if body weight is less than 40 kg) without a strong CYP3A4 inhibitor.

Regarding Phase 2 data, as of July 24, 2023, 94 patients aged 1.3–75 years with R/R KMT2A-r acute leukemia had received at least one dose of the study drug and were included in the safety analysis. Treatment discontinuation due to TRAEs was infrequent at 6%. The most common TRAEs included nausea (27.7%), DS (26.6%), and QTc prolongation (23.4%) [87].

The interim analysis for the efficacy population (*n* = 57) in Phase 2, which included patients with centrally confirmed KMT2A acute leukemia, ≥5% blasts in the bone marrow at baseline, and those who had started treatment concurrently or before the 38^th^ adult AML efficacy evaluable patient, met the primary endpoint. The combined CR+CRh rate was 22.8% (13/57; 95% CI 12.7–35.8), achieving a one-sided P value of 0.0036, with 70% of patients achieving a negative measurable residual disease status. The ORR was 63.2%, and the CRc was 43.9%.

Regarding Phase 2 data in the pediatric population, a total of 36 patients older than 30 days were included (23 in safety population and 13 in efficacy population). The CR+CRh rate was 23% (3/13; 95% CI 5.0–53.8), with 67% achieving negative measurable residual disease status. The ORR was 46% and the CRc was 38.5% [88].

The promising preliminary results of AUGMENT-101 prompted the opening of AUGMENT-102 (NCT05326516), a phase 1 trial investigating revumenib in combination with chemotherapy in patients with *KMT2A*-r or *NPM1*-m acute leukemias. Chemotherapy in the first portion of the cycle is hypothesized to reduce the risk of DS and promote synergistic lethality in AML cells [89]. Additional pediatric-specific investigation of revumenib with chemotherapy is ongoing via the Children’s Oncology Group AALL2121 clinical trial (NCT05761171) for young children with relapsed/refractory *KMT2A*-rearranged ALL and planned via other consortia for other children with relapsed/refractory ALL or AML with relevant genetic alterations.

Preliminary data from AUGMENT-101, which investigates the feasibility of maintenance therapy after HSCT with revumenib in pediatric patients, show promising safety and efficacy outcomes. However, further analysis is needed to determine optimal treatment durations and safety profiles [90].

#### Ziftomenib

The KO-MEN-001 trial (NCT04067336), the first-in-human phase 1/2 trial of ziftomenib (KO-539, Kura Oncology), aimed to determine the drug’s RP2D and assess its safety and efficacy in adult patients with relapsed or refractory AML. In Phase 1a, ziftomenib was generally well-tolerated, with no dose-related adverse events; however, there were 2 dose-limiting toxicities: pneumonitis in 1 patient at the 400-mg dose and DS in 1 patient at the 1000-mg dose. There were no reports of QTc prolongation. Per protocol, the DS at the 1000-mg dose was considered a dose-limiting toxicity and resulted in de-escalation. The ziftomenib dose was reduced to 600 mg, which became the RP2D. At 600 mg, 20% of patients with *NPM1*-m disease and 38% of those with *KMT2A*-r disease experienced DS; a quarter of patients with *NPM1*-m and 25–30% of those with *KMT2A*-r had grade 3 or higher DS [91].

The pharmacokinetics and clinical activity of ziftomenib did not appear to be affected by the co-administration of a CYP3A4 inhibitor.

In Phase 1b, the 600-mg daily dose showed promise, with 16.7% of patients having a CR/CRh, leading to the discontinuation of the 200-mg cohort. Subsequent data collected up to October 2022 consistently favored the 600-mg dose, particularly in patients with *NPM1*-m disease, whose CR/CRh rate was 21.1%. By April 2023, these patients’ CR/CRh rate had risen to 35% [91]. There were no clear dose-related adverse events, and the most significant concern was DS, which was observed in 20% of AML patients, including one with grade 5 DS.

The trial’s pharmacokinetic and pharmacodynamic data showed a positive relationship between ziftomenib exposure and anti-leukemic response and supported the RP2D of 600 mg. In conclusion, ziftomenib at 600 mg offers promising efficacy with manageable safety for relapsed or refractory AML, potentially representing a breakthrough for this challenging patient population. Pediatric-specific investigation of ziftomenib is ongoing.

### Other agents in development

#### JNJ-75276617

Another relevant Phase I trial is 75276617ALE1001 (NCT04811560), with patients receiving the menin inhibitor JNJ-75276617. The cohort includes adult patients with refractory relapsed acute leukemia with KMT2A-r or NPM1 mutations. A subsequent amendment permitted enrollment of children aged 12 years and older. The most frequent TRAE was DS (14%). The RP2D has not been established yet [92]. NCT05453903 is a Phase 1b ongoing trial that combines JNJ-75276617 with venetoclax and azacytidine in patients with AML with KMT2Ar or NPM1 mutations. The safety dataset included 45 patients, and the efficacy dataset included 21 patients. No DS and DLTs were reported, and the most common side effects were GI symptoms (nausea, emesis) and thrombocytopenia. The overall response rate (ORR) was reported at 86% [93].

#### DSP-5336

A first-in-human Phase 1/2 study involving the menin inhibitor DSP-5336 was recently presented at the European Hematology Association (EHA) and included two parallel arms: Arm A without concomitant anti-fungal azoles, and Arm B with azoles. The median age of participants was 63.0 years. Patients had received a median of 3 prior treatments, including prior allogeneic stem cell transplant. Results from a cohort of 58 patients with either KMT2Ar or NPM1 mutations revealed an ORR of 45% in patients who received doses higher than 140 mg BID [94]. No DLTs were observed. Three cases of possible DS were documented, with no treatment discontinuations, intensive care unit (ICU) stays, or mortality. This study has also been amended to allow the enrollment of children aged 12 years and older.

#### BMF-219

The COVALENT-101 (NCT05153330) is a Phase I trial of BMF-219, an oral covalent menin inhibitor. The study analyzed dose-escalation in patients taking or not taking CYP3A4 inhibitors. The preliminary data from a cohort of adults with refractory or relapsed acute leukemia was presented recently. Common TRAEs were emesis and DS. There were no dose-limiting toxicities and no discontinuation of treatment due to toxicities [95].

BMF-219 has sparked controversy due to differing response profiles and gene expression data compared to established menin inhibitors. Recent studies have questioned its classification as a menin inhibitor highlighting ongoing uncertainty [96].

Other compounds have demonstrated promising activity in preclinical models and are currently progressing toward clinical development [97].

### Resistance mechanisms

**Fig. 3:**
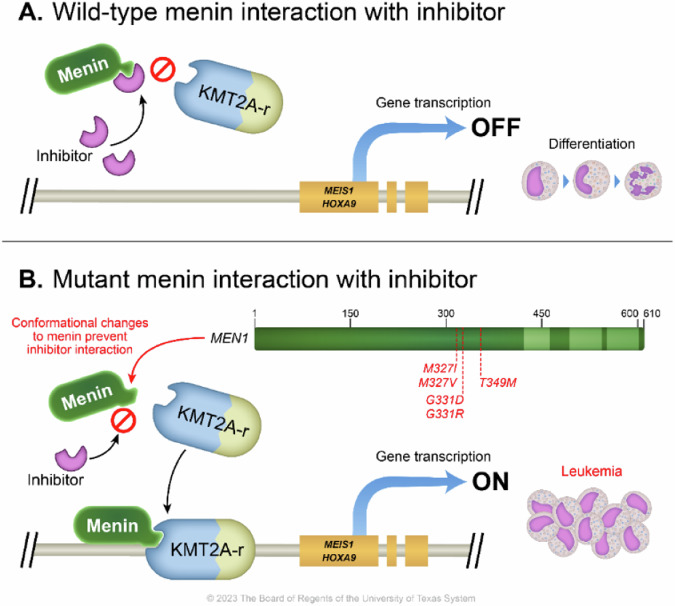
Mechanisms of resistance to menin inhibition. **A** Illustrates the mechanism of menin inhibition, showing how it interferes with the interaction between menin and KMT2A. This in turn prevents the aberrant expression of HOX genes and *MEIS1*, leading to the release of the differentiation block. **B** Somatic mutations in *MEN1* hinder the displacement of the menin-MLL1 protein complex, thereby preventing the gene expression changes necessary to inhibit leukemic cell self-renewal and induce myeloid differentiation.

Recently, Perner et al. identified somatic mutations in the *MEN1* gene, which encodes the menin protein, at the interface of revumenib’s interaction with menin [98]. These mutations were observed in patients who developed resistance to menin inhibitors while receiving the therapy, but were not detected in the pre-treatment AML cells (Fig. 3). Of the 31 patients tested, 12 (39%) showed the presence of these resistance mutations, often within 2 treatment cycles of monotherapy. In model systems of both *KMT2A*-r and *NPM1*-m disease, *MEN1* mutations hindered the displacement of the menin-MLL1 protein complex from chromatin at critical target genes. Consequently, they prevented the gene expression changes necessary to inhibit leukemic cell self-renewal and induce myeloid differentiation. In response to the resistance observed with first-generation menin inhibitors, researchers are actively investigating second-generation compounds that specifically target MLL1 binding without interacting with mutated residues in MEN1. Menin inhibitor-based synergistic combinations with other agents, including venetoclax, FLT3 inhibitor, CDK4/6 inhibitor, BET inhibitor, DOT1L inhibitor, LSD1 inhibitor, Ikaros protein degrading molecular glues, BRG1/BRM inhibitor, and CBP/p300 inhibitor, have been reported. These combinations could potentially overcome the emergence of AML cells carrying menin mutations and the development of menin inhibitor resistance [98–107]. Notably, early data suggests that resistance to menin inhibitor could also be non-genetic (without menin mutations), likely based on epigenetic/adaptive mechanisms [108]. Here again, the menin inhibitor-based synergistic combinations prevent or abrogate the epigenetic/adaptive mechanisms of menin inhibitor resistance. These promising observations underscore the potential of developing alternative strategies to overcome resistance to menin inhibitors. Ongoing studies will enhance our understanding of other resistance pathways, such as acquired mutagenesis of essential, non-driver epigenetic regulators, and pave the way for novel therapeutic approaches. The significance of certain MEN1 mutations should be approached with caution as they are subclonal, leading to a limited understanding of their relevance. Consequently, when combined with chemotherapy, these mutations may not hold substantial significance.

The Leukemia and Lymphoma Society’s PedAL/EUpAL plans to conduct phase 1–2 clinical trials of menin inhibitor-based strategies in pediatric patients with relapsed or refractory AML or ALL carrying alterations in *KMT2A*, *NPM1*, or *NUP98*. These trials aim to establish safe and potentially effective pediatric dosages of ziftomenib in combination with appropriate multi-agent chemotherapy. Other Menin Inhibitors are exploring their effectiveness in pediatric patients by expanding adult study criteria to include individuals as young as 12, accelerating our development efforts significantly (Table 1).

**Table 1:** Current and future trials of menin inhibitors in pediatric patients.

Current					
Clinical trial/sponsor	Phase	Treatment	Biomarkers	Age eligibility	Location
AUGMENT-101 (NCT04065399)	1/2	Revumenib monotherapy	*KMT2A*-r, *NPM1*-m, *NUP98*-r	All ages; AML and ALL	Multisite- U.S. and Europe
AUGMENT-102 (NCT05326516)	1	Revumenib + FLA	*KMT2A*-r, *NPM1*-m, *NUP98*-r	All ages AML and ALL	Multisite- U.S. and Europe
SAVE (NCT05360160)	1/2	Revumenib + Venetoclax+ ASTX727	*KMT2A*-r, *NPM1*-m, *NUP98*-r (Frontline and Relapse)	≥12 years AML and MPAL	MD Anderson Cancer Center
TINI 2 (NCT05848687)	1	Ziftomenib + Multiagent	*KMT2A*-r	Infant ALL	Stanford University
AALL2121 / COG (NCT05761171)	2	Revumenib + Multiagent	*KMT2A-r*	30 days to 6 years ALL and MPAL	Multisite US
(NCT04811560)	1	JNJ-75276617 + Multiagent	*KMT2A*-r, *NPM1*-m, *NUP98*-r, *NUP214*-r	>12 years. AML and ALL	Multisite- US and Europe
(NCT06177067)	1	Revumenib + Venetoclax + Azacytidine.	*KMT2A*-r, *NPM1*-m, *NUP98*-r	1-30yo. AML and ALL	St Jude Children’s Hospital
FUTURE					
	1	Ziftomenib + Venetoclax + Azacytidine	*KMT2A*-r, *NPM1*-m, *NUP98*-r	<40 yo; AML and ALL	MD Anderson Cancer Center
APAL2020K ITCC-101/COG/PEDAL	1	Ziftomenib + FLA	*KMT2A*-r, *NPM1*-m, *NUP98*-r	<18 yo; AML and ALL	Multisite- U.S. and Europe
	1	Ziftomenib + Venetoclax + Gemtuzumab	*KMT2A*-r, *NPM1*-m, *NUP98*-r, *UBTF-ITD*	<40 yo; AML and ALL	MD Anderson Cancer Center
	1	Revumenib + AD + Gemtuzumab	*KMT2A*-r, *NPM1*-m, *NUP98*-r, *UBTF-ITD*	<40 yo; AML and ALL	MD Anderson Cancer Center

*Early relapse is defined as relapse within one year of first complete remission.

*FLA* fludarabine and cytarabine, *IDA* idarubicin, *A* cytarabine, *D* daunorubicin.

## Opportunities to accelerate progress in pediatric oncology

### International collaborations

**Fig. 4:**
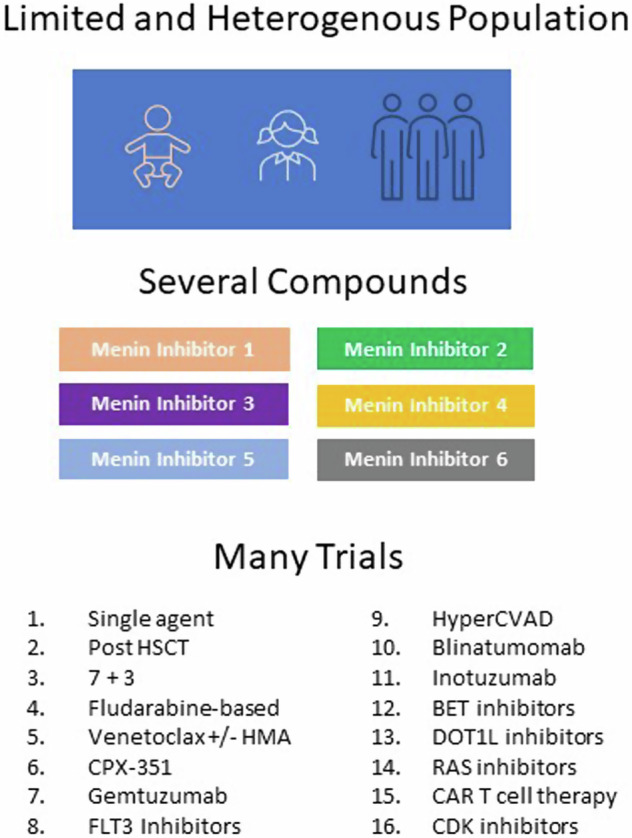
Future combinations of menin inhibitors in pediatric leukemia. Challenges in a diverse and small population.

Global collaboration in pediatric drug development is paramount, necessitating a departure from relying solely on mature adult efficacy data. Menin inhibitors hold immense promise for addressing unmet needs in various pediatric leukemia subtypes, including infant KMT2A-r ALL, high-risk KMT2A-r AML, NUP98-r AML, and others. However, enrolling pediatric patients in trials presents significant challenges, mainly due to the small number of affected individuals. This limitation impedes rapid insights into various aspects of menin inhibitors, including side effects, efficacy across leukemia subtypes and fusion partners, variability in inhibitor efficacy, resistance mechanisms, and effective combinations pre and post HSCT (Fig. 4). A unified global development plan for menin inhibitor therapy necessitates collaboration among international groups. Without such coordination, progress will stagnate, delaying its integration into frontline treatments for decades.

Through international collaboration, we can streamline efforts by eliminating redundant studies in this already limited heterogeneous population, expediting recruitment, enhancing data sharing, and validating results essential for regulatory compliance. This approach also has the potential to accelerate the approval of menin inhibitors across diverse jurisdictions, ultimately benefiting all patients regardless of geographic location or available resources.

**Fig. 5:**
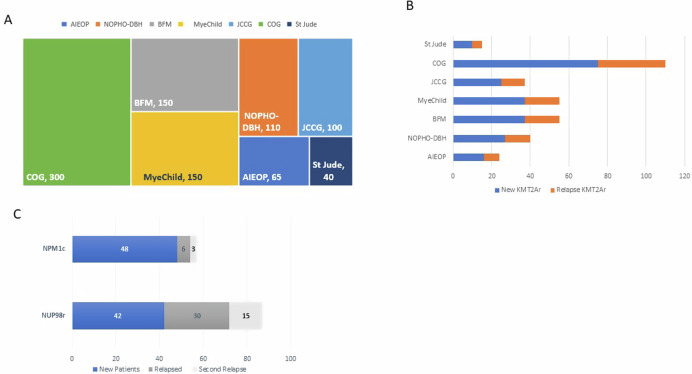
Estimated annual incidence of newly diagnosed and recurrent AML in pediatric patients globally and in the US. **A** International study groups’ estimated annual incidences of newly diagnosed pediatric AML [78]. AIEOP (Associazione Italiana Ematologia Oncologia Pediatrica; Italy), NOPHO-DB-SHIP (Nordic Society of Pediatric Hematology and Oncology; [Denmark, Finland, Iceland, Norway, and Sweden, Latvia, Lithuania, Estonia, the Netherlands, Belgium, Hong Kong, Spain, Portugal and Israel]), BFM (Berlin-Frankfurt-Münster Oncology; Austria, Czech Republic, Poland, Hungary, Slovakia, Slovenia, Croatia, Serbia, Bulgaria, Romania, and Turkey), MyeChild (United Kingdom), JCCG (Japan Children’s Cancer Group; Japan), COG (Children’s Oncology Group; United States and Canada), St. Jude (United States), **B** Estimated annual incidence of newly diagnosed and recurrent *KMT2A*-r AML among pediatric patients. **C** Estimated annual incidence of newly diagnosed and relapsed NPM1c and *NUP98*-r AML among pediatric patients in COG studies.

For instance, Menin inhibitors hold the potential to reshape the treatment paradigm for up to 60% of AML patients worldwide. However, when considering the global incidence of pediatric AML cases, encompassing all genotypes, the number is less than 1000 (Fig. 5A). As we delve into smaller subsets, especially those facing relapse, the importance of collaboration becomes increasingly apparent (Fig. 5B, C).

### Collaboration with adult leukemia

In parallel, forging partnerships with adult counterparts is critical to parallel our drug development. Adults often have quicker access to drugs, which can significantly expedite research progress. Expanding our inclusion criteria to include individuals as young as 12 could offer significant benefits in pediatric research. If a menin inhibitor study combining adults and children reveals any toxicities or limited responses, the insights gained can help us avoid similar combinations in future pediatric studies, ultimately saving time and resources. On the other hand, if an adult trial including children shows promising results, we can delve deeper into its implications or expand its registration scope. Such changes to trial design could pave the way for exploring these drugs in even younger age groups, offering hope for better pediatric treatment outcomes.

While randomized studies are typically preferred for generating scientific insights, the rarity of pediatric leukemia subtypes and multiple products in the same class suggest a shift towards well-structured single-arm trials. A recommended strategy involves a sequential approach, building upon previous studies’ findings on safety, efficacy, and optimal dosing. This worldwide partnership between pediatric and adult teams could lead to more informed decisions about further development and potential regulatory approval.

This initiative could begin with establishing a structured network through cancer centers that integrate pediatric and adult oncologists. Investigator-initiated and sponsor trials would be conducted simultaneously in both adult and pediatric settings within each institution, managed by independent primary investigators and research coordinators. This approach would ensure precise oversight and customized monitoring for each age group. Leveraging the infrastructure of established groups like COG and EORTC, could yield significant benefits. Careful management is crucial to avoid bureaucratic obstacles that might otherwise impede the speed of drug development and clinical trial implementation.

## Conclusions

In conclusion, realizing the full potential of menin inhibitors in pediatric leukemia treatment demands global cooperation. Overcoming enrollment hurdles, sharing data, and forging partnerships are vital for accelerating progress and enhancing outcomes globally. A joint effort between pediatric and adult oncology is essential for fulfilling the promise of menin inhibitors and meeting unmet needs in pediatric oncology. Our focus should now shift towards optimizing the utilization of existing drugs. The international collaboration among our authors signifies a significant step towards this goal, ushering in a new era of cooperation with adults and innovation in pediatric leukemia therapeutics.
